# Germline-Restricted Chromosome (GRC) in Diploid and Polyploid Spermatocytes of the Eurasian Bullfinch, *Pyrrhula pyrrhula* (Fringillidae, Passeriformes, Aves)

**DOI:** 10.3390/ani15233394

**Published:** 2025-11-24

**Authors:** Ekaterina Grishko, Lyubov Malinovskaya, Katerina Tishakova, Pavel Borodin

**Affiliations:** 1 Laboratory of Genome Structure and Function, Novosibirsk State University, 630090 Novosibirsk, Russia; grishko@bionet.nsc.ru (E.G.); l.malinovskaia@g.nsu.ru (L.M.); tishakova@mcb.nsc.ru (K.T.); 2 Laboratory of Recombination and Segregation Analysis, Institute of Cytology and Genetics, 630090 Novosibirsk, Russia; 3 Laboratory of Diversity and Evolution of Genomes, Institute of Molecular and Cellular Biology, 630090 Novosibirsk, Russia

**Keywords:** germline-restricted chromosome (GRC), synaptonemal complex, chromosome synapsis, tetraploid spermatocyte, MLH1, H3K9me3, SYCP3

## Abstract

The germline-restricted chromosome (GRC) is a unique and enigmatic element found exclusively in the germ cells of passerine birds, with its function and evolutionary dynamics still largely unresolved. This study utilizes cytogenetic analysis of the Eurasian bullfinch (*Pyrrhula pyrrhula*) to explore the meiotic behavior of the GRC. We report the novel discovery of naturally occurring tetraploid and octoploid spermatocytes in this species. Remarkably, in these polyploid cells, the GRC exhibited normal meiotic processes, including full synapsis and recombination. Recombination was restricted to the H3K9me3-negative proximal half of the GRC bivalent, implicating a chromatin-based regulation mechanism. The standard chromosome set in the polyploid cells showed orderly chromosome synapsis. The number of recombination nodules in tetraploid and octoploid nuclei was approximately equal to the standard value for diploids multiplied by 2 and 4, respectively. These findings suggest that polyploidy does not completely hinder meiotic progression in birds and offer new insights into GRC regulation during meiosis.

## Simple Summary

Programmed DNA elimination is a process found in some animals, where specific genomic regions are removed from body cells but retained in reproductive cells. In passerine birds, an additional chromosome called the Germline-Restricted Chromosome (GRC) exists exclusively in reproductive cells. We discovered that in rare Eurasian bullfinch cells with doubled or quadrupled chromosome numbers, the GRC, along with the standard chromosomes, pairs and recombines normally. This unusual behavior may explain how some birds produce unreduced sperm, leading to rare triploid offspring.

## 1. Introduction

Programmed DNA elimination is a phenomenon observed in various unicellular and multicellular eukaryotes and has recently been discovered in birds. In all passerine birds studied so far, germ cells possess an additional germline-restricted chromosome (GRC), which is eliminated from somatic cells during early embryonic development [1,2]. The GRC contains amplified and modified copies of genes derived from the standard genome, some of which are expressed in both male and female gonads [3,4,5,6,7,8].

GRC exhibits interspecies variation in its size and genetic content. Fluorescent in situ hybridization (FISH) with whole-chromosome probes revealed a surprisingly low degree of homology between GRCs of different species, indicating rapid evolutionary turnover of their genetic composition [2].

Typically, female germ cells carry two copies of the GRC, which show orderly chromosome synapsis and recombination during meiotic prophase. In contrast, male germ cells usually possess a single copy of the GRC that undergoes heterochromatinization during meiosis I. It is subsequently eliminated as a micronucleus after the first meiotic division [9,10]. While GRC is predominantly maternally inherited, rare cases of paternal transmission have been documented in the zebra finch [11].

Mosaicism in GRC copy number was described in ten out of the 76 males from 27 species examined. These ten individuals comprised seven pale martins (*Riparia diluta*), and one individual each of great tit (*Parus major*), European pied flycatcher (*Ficedula hypoleuca*), and black-headed munia (*Lonchura atricapilla*). Some spermatocytes contained two and, in one case, three copies of GRC [8]. In all cases except the last, the GRC either did not synapse at all or engaged in partial synapsis at the chromosome ends. The only case of complete synapsis was observed in a male pale martin with three copies of GRC (a bivalent and a univalent). In one of the mosaic pale martins, recombination nodules were occasionally observed at the ends of partially synapsed GRCs [10]. In all examined females with two GRCs, recombination was restricted to chromosome ends [2]. It is not clear what causes the mosaicism—whether biparental inheritance of GRC, nondisjunction during maternal meiotic divisions, or mitotic divisions of the primordial spermatogenic cell. Similarly, the reasons underlying the sexual differences in synapsis of two GRC copies are also unresolved.

In this study, we analyze synapsis, recombination, and epigenetic modifications of the GRC in diploid and polyploid spermatocytes of the Eurasian bullfinch (*Pyrrhula pyrrhula*). The standard karyotype of the bullfinch, like many passerines, comprises 41 chromosome pairs in somatic cells (2n = 82) [12]. However, the proximal part of its GRC is H3K9me3-negative, whereas the GRC of all other songbirds examined is completely H3K9me3-positive. Bullfinch polyploid germ cells are particularly interesting because they may contain multiple GRC copies. This unique system provides a novel context for investigating how this rapidly evolving chromosome behaves under conditions of whole-genome duplication in germ cells.

## 2. Materials and Methods

### 2.1. Specimens

Testes from two sexually mature male bullfinches were obtained from birds admitted with fatal injuries to the Wildlife Rehabilitation Center in Novosibirsk during April–May 2023. Handling of the birds and euthanasia were conducted in accordance with national regulations on the housing and use of laboratory animals. Euthanasia was performed using an overdose of isoflurane.

The study is reported in accordance with the ARRIVE guidelines (Animal Research: Reporting of In Vivo Experiments) (accessed at https://arriveguidelines.org on 11 September 2025). The protocol was reviewed and approved by the Bioethics Committee of the Institute of Cytology and Genetics, Siberian Branch of the Russian Academy of Sciences (protocols #45/2 of 10 January 2019 and #114 of 17 December 2021).

### 2.2. Synaptonemal Complex (SC) Spreading and Staining

Chromosome preparations were made using the drying-down technique with fixative vapor exposure [13]. For electron microscopy, slides were stained with silver nitrate [14]. The slides were then immersed in a 1% solution of plastic dissolved in chloroform for 5 s to create a thin transparent film on the glass surface, which was subsequently dried at room temperature for two days.

After examination under a light microscope, the plastic films were transferred onto specimen grids and analyzed using a JEM1400 transmission electron microscope (Jeol, Tokyo, Japan) at an accelerating voltage of 80 kV.

Immunostaining was performed according to the protocol described by Anderson et al. [15], using the antibodies listed in Table 1.

Slides were incubated in a humid chamber overnight at +4 °C with the primary antibodies and for one hour at 37 °C with the secondary antibodies. To prevent photobleaching, slides were mounted in Vectashield medium (Vector Laboratories, Newark, CA, USA; catalog no. H-1000-10).

To analyze the distribution of MLH1 foci relative to H3K9me3 staining along the GRC bivalents in tetraploid cells, we performed sequential multicolor immunostaining. First, slides were immunostained with anti-SYCP3, anti-MLH1 and anti-centromere antibodies, and nuclei of interest were captured, with their coordinates recorded. Subsequently, the same slides were washed in PBST for 5 min and immunostained with anti-H3K9me3 antibodies. The previously captured nuclei were then relocated using the recorded coordinates and recaptured.

### 2.3. Generation of DNA Probe for the Bullfinch GRC

The DNA probe for the bullfinch GRC was generated by microdissecting five copies of micronuclei from conventionally prepared meiotic chromosome spreads, as described by Torgasheva et al. [2]. The spreads were stained with 0.1% Giemsa solution (Sigma-Aldrich, Saint Louis, MO, USA) for 3–5 min at room temperature. DNA from microdissected micronuclei was amplified and labeled with biotin-11-dUTP (Sigma-Aldrich, Saint Louis, MO, USA) using the GenomePlex Whole Genome Amplification Kit (Sigma-Aldrich, Saint Louis, MO, USA; catalog no. WGA1).

### 2.4. FISH with GRC DNA Probe

Fluorescence in situ hybridization (FISH) with the bullfinch GRC DNA probe was performed on immunostained SC spreads according to the standard protocol [16] with some modifications. Briefly, slides were washed in 2 × SSC for 5 min and then rehydrated through a graded ethanol series. Hybridization mixture (32 µL) contained hybridization buffer (50% formamide, 2 × SSC), 0.2% Tween 20, and 40 ng of labeled probe. RNAseA-treated slides were denatured in 70% formamide with 2 × SSC at 72 °C for 3 min. The probe was denatured at 95 °C for 5 min. Hybridization was carried out at 39 °C overnight in a humid chamber. Biotin-labeled GRC probe was detected using avidin-FITC (Vector Laboratories, Newark, CA, USA; catalog no. A-2001-5) and anti-avidin-FITC antibodies (Vector Laboratories, Newark, CA, USA). Slides were mounted in Vectashield medium with DAPI (Vector Laboratories, Newark, CA, USA; catalog no. H-1200-10).

### 2.5. Image Analysis

Images of synaptonemal complex (SC) spreads after immunostaining and FISH were captured using a CCD camera installed on an Axioplan 2 compound microscope (ZEISS, Oberkochen, BW, Germany) equipped with filter cubes #49, #43HE (ZEISS, Germany) and #SP101, #SP104v1 (Chroma, Bellows Falls, VT, USA) using ISIS4 software (METASystems GmbH, Altlussheim, BW Germany). Brightness and contrast of all images were enhanced using Corel PaintShop Photo Pro X6 software (Alludo, Ottawa, ON, Canada).

We classified nuclei as zygotene if less than 90% of the axial elements of the SC were synapsed; as early pachytene if synapsis involved more than 90% and less than 100% of SC length; and as mid-late pachytene if nuclei had complete synapsis (excluding GRCs and some regions of the quadrivalents) (Supplementary Figure S1).

Nuclei were classified as polyploid only if they met all the following conditions:

(i) The nucleus is well-isolated from other nuclei, exhibits a continuous regularly shaped outline and all SCs within the nucleus are at the same stage of meiotic prophase.
(ii) At the early and mid-late pachytene stage, the nucleus contains more than 70 SCs (approximately double the diploid chromosome number). At the zygotene stage, the nucleus contains two synapsed or closely located GRCs.

We identified the GRCs by the brightest FISH signal of the GRC DNA probe or its distinct pattern of H3K9me3 staining and centromeric index.

The length of the GRC’s SC and arms of quadrivalents was measured in micrometers (µm). MLH1, centromere and H3K9me3 positions were recorded using MicroMeasure 3.3 software [17]. All raw measurements are provided in Supplementary Table S1.

### 2.6. Statistical Analysis

Descriptive statistics were performed using Statistica 6.0 (StatSoft Inc., Tulsa, Ok, USA). Mean ± standard deviation (SD) values are reported in the text.

## 3. Results

**Figure 1:**
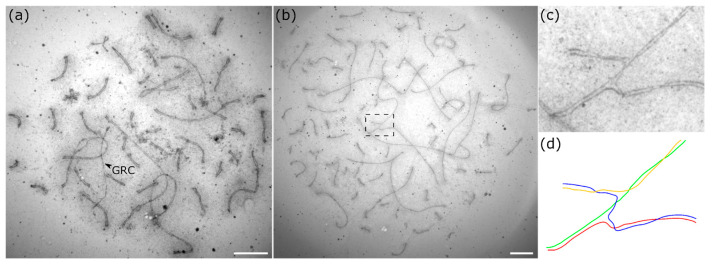
Electron microphotographs of diploid (**a**) and tetraploid (**b**) pachytene spermatocytes of the bullfinch after silver staining. (**c**) Enlarged photo and (**d**) schematic representation of the region (dotted box in **b**) of switching pairing partners in the quadrivalent. The arrowhead indicates the GRC univalent. Scale bar—5 µm.

**Figure 2:**
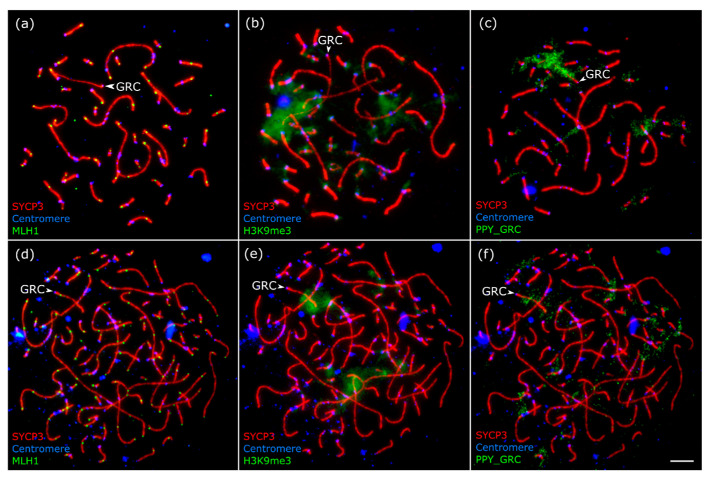
Microphotographs of a diploid (**a**–**c**) and a tetraploid (**d**–**f**) pachytene spermatocytes of the bullfinch after immunostaining and FISH with the GRC-specific probe (PPY_GRC) (**c**,**f**). Arrowheads indicate the GRCs. (**d**–**f**) The same tetraploid pachytene spermatocyte stained with antibodies to SYCP3 (**d**–**f**), centromere proteins (**d**–**f**), MLH1 (**d**), and H3K9me3 (**e**). Scale bar—5 µm.

In diploid pachytene spermatocytes, the GRC appeared as a univalent evenly stained with silver nitrate (Figure 1a) or labeled by SYCP3 antibodies (Figure 2a). The univalent was not involved in ectopic synapsis with the chromosomes of the basic set. It did not form foldbacks of the self-synapsis. We did not observe signals of MLH1 (protein of late recombination nodes) on it at the mid-late pachytene stage, when each chromosome of the basic set contained at least one signal (Figure 2a).

Previously, in SC preparations from one bullfinch individual, we identified 35 tetraploid and one octoploid spermatocyte [12]. Here we examine and describe them in more detail. Among the tetraploid cells, 5 were at the zygotene stage, 17 at the early pachytene stage, and 13 were at the mid-late pachytene stage (Supplementary Figure S1). Most of the mid-late pachytene tetraploid spermatocytes contained about 83 bivalents (Figure 1b and Figure 2d).

**Figure 3:**
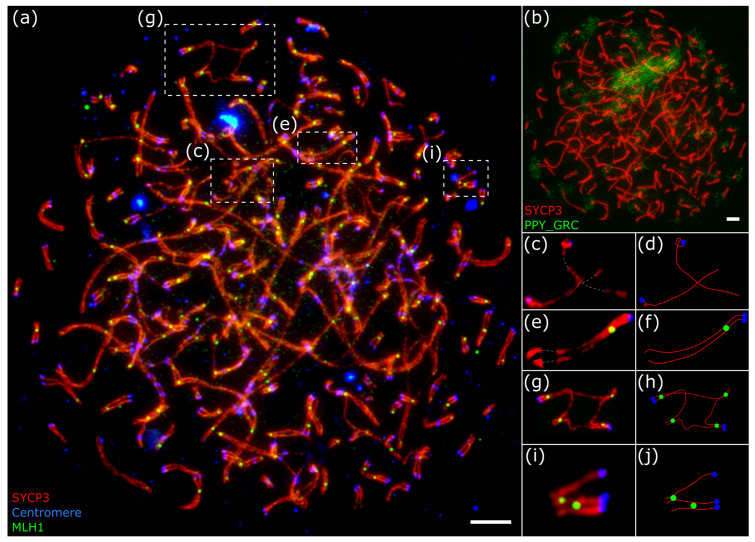
Microphotographs of the octoploid pachytene spermatocyte of the bullfinch after immunostaining (**a**) and FISH with the GRC-specific probe (PPY_GRC) (**b**). (**c**–**j**) Enlarged photo and schematic representation of the asynapsed GRC (**c**,**d**), synapsed GRC (**e**,**f**), quadrivalent (**g**,**h**) and trivalent (**i**,**j**). Scale bar—5 µm.

In six pachytene nuclei, we observed one quadrivalent (Figure 1c,d and Figure 3g,h, Supplementary Figures S1 and S2); in one nucleus–two quadrivalents; and in two nuclei–three quadrivalents.

To distinguish homologously synapsed quadrivalent configurations from structures that might mimic them, we conducted detailed measurements. Interlocking or juxtaposed configurations produced by non-homologous bivalents and multivalents, resulting from non-homologous chromosome synapsis, can resemble genuine quadrivalents. Therefore, we measured the lengths of all four arms in 14 well-traceable configurations (Figure 1b and Figure 3g; Supplementary Figures S1 and S2; Supplementary Table S1). We assumed that the opposite arms of genuine quadrivalents should be approximately equal, whereas in other configurations the ‘arms’ may not match. The correlation between the lengths of opposite arms was quite high (R = 0.99, n = 28, t = 35, *p* < 0.0001). These data confirm that the quadrivalent configurations observed in tetraploid nuclei were indeed homologous quadrivalents.

The remaining 21 pachytene nuclei did not contain quadrivalents. Multivalent configurations were never observed in the diploid spermatocytes. Different macro- and micro-SCs were involved in these configurations. All quadrivalents detected had at least one MLH1 focus at each of the four synapsed side arms (Figure 3g,h). In the octoploid spermatocyte, in addition to many quadrivalents, we found several trivalents (Figure 3i,j).

The average number of MLH1 foci was roughly double in tetraploid cells (90.5 ± 6.8, n = 12 in early pachytenes; 98 ± 3, n = 9 in mid-late pachytenes) and quadruple in the single octoploid cell (193, n = 1) of that in the diploid spermatocytes at the mid-late pachytene of the same individual bullfinch (50.9 ± 1.9 MLH1 foci, n = 59) [12] (see supplementary material https://www.mdpi.com/article/10.3390/ani13233624/s1 (accessed on 10 October 2025), individual Eurasian bullfinch 1M-2023) (Figure 2a,d and Figure 3a).

In polyploid spermatocytes, the GRCs often form completely synapsed bivalents that are visually indistinguishable from the bivalents of the basic chromosome set (Figure 2d, Supplementary Figure S1). They can only be visualized by the specific pattern of FISH (Figure 2f) or by H3K9me3 immunolocalization (Figure 2e).

The FISH probe PPY_GRC produced a strong signal at the GRC and weak signals at the centromeric regions of some macrobivalents and at the centromeric and telomeric regions of some microbivalents (Figure 2c,f).

H3K9me3 is a mark of constitutive heterochromatin. Grishko et al. [12] reported that, in male bullfinches, H3K9me3 antibodies bind to the pericentromeric region and the distal half of the GRC (Figure 2b), while in other passerine species examined, the antibodies labeled the entire chromosome. In tetraploid spermatocytes, the GRC exhibits the same pattern (Figure 2e).

Using FISH, we detected the GRC in 23 tetraploid spermatocytes and in the single octoploid spermatocyte (Figure 2f and Figure 3b). At the zygotene stage, the GRC was mostly asynapsed in the majority of tetraploid cells. By early pachytene, it became fully synapsed in most cells. By mid-late pachytene, the GRC was fully synapsed in almost all cells. In the octoploid spermatocyte, two GRC copies were partially synapsed with each other, while the other two were closely positioned but asynapsed (Figure 3c–f).

**Figure 4:**
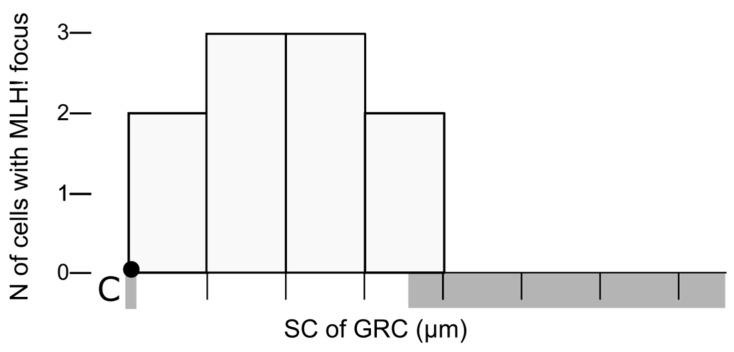
Distribution of MLH1 signals along the bullfinch GRC bivalent. The *x*-axis represents GRC length (1 µm per division). The height of each bar indicates the number of cells in which the MLH1 signal localizes to that segment. The centromere is marked by a black dot. Chromosomal regions labeled with H3K9me3 are shown in gray. n = 10.

The average SC length of GRC bivalents, measured in 14 tetraploid nuclei, was 7.6 ± 0.9 µm, while the average length of the GRC univalent, measured in 38 diploid nuclei, was 12.0 ± 2.4 µm. Comparable length differences have been reported between the GRC bivalents and univalents in the oocytes of the sand martin [10]. All identified synapsed GRC bivalents (n = 12) contained a single MLH1 focus. In 10 of them, it was possible to determine the location of the MLH1 relative to the centromere: in all cells, the MLH1 focus was the proximal H3K9me3-negative part (Figure 4).

## 4. Discussion

### 4.1. A Curious Normality of Chromosome Pairing and Recombination in the Polyploid Bullfinch Spermatocytes

Notable features of tetraploid spermatocytes in the bullfinch are the orderly pairwise chromosome synapsis and the proportional increase in the recombination observed in the basic chromosome set. Quadrivalents were rare, interlockings were absent, and the recombination rate in tetraploid cells was almost exactly double that of diploids. The predominant formation of bivalents, rather than multivalents, suggests exceptionally high fidelity of strictly homologous pairing in these cells. In the rare quadrivalents, each pairing segment contained at least one recombination nodule, indicating that these were stable, recombined configurations committed to proper chromosome disjunction rather than transient intermediates in synaptic adjustment. By contrast, the octoploid spermatocyte showed numerous synaptic abnormalities, including many quadrivalents, trivalents, and univalents, despite a normal overall recombination rate.

This apparent normality implies that tetraploid germ cells in bullfinches (and possibly other bird species) can progress through male meiosis without arrest, potentially producing viable sperm. Polyploidy is typically lethal in mammals and often results in embryonic mortality in birds [18,19]. Nevertheless, viable triploid individuals have been reported in the domestic chicken, the Kentish plover, the blue-and-yellow macaw, and the zebra finch, although all tested triploids were sterile [20,21,22]. Triploids may arise via parthenogenesis or through fusion of reduced and unreduced gametes.

Tetraploid spermatocytes have also been observed in mammals, mostly as isolated incidents [23,24,25,26], but multiple occurrences have been reported in humans and mice [27,28,29]. Endoreduplication before meiosis has been proposed as a likely cause of the origin of the tetraploid meiocytes [24]. In the bullfinch studied, the exact monoclonal or polyclonal origin of tetraploid spermatocytes could not be deduced. Within the framework of the more parsimonious monoclonal hypothesis, the endoreduplication might have occurred as early as the sixth premeiotic germ cell division (since 2^6^ = 64 > 42).

### 4.2. GRC Behavior in Polyploid Bullfinch Spermatocytes

Synapsis and recombination of GRCs in polyploid spermatocytes have not been reported previously, though GRC bivalents were seen in spermatocytes of the pale martin and European pied flycatcher with variable GRC numbers [8,10]. In all pachytene spermatocytes of these specimens, the behavior of the GRC bivalent differed significantly from those of the basic chromosome sets. The completely synapsed bivalent was observed in only one spermatocyte with the third GRC present as a univalent GRC. Otherwise, the two GRCs either did not synapse or synapsed only at the chromosome ends, exhibiting a single MLH1 focus at each end [8,10].

GRC bivalents are typical for passerine pachytene oocytes, which normally contain two GRCs. Synapsis and recombination of two GRCs in females have been described in several species (zebra finch, Bengalese finch, European pied flycatcher, great tit, sand martin, pale martin, and barn swallow). They are characterized by full synapsis with one or two MLH1 foci at bivalent ends [9,10,30,31,32]. GRC behavior in tetraploid bullfinch spermatocytes closely resembles that in oocytes of other species examined rather than diploid spermatocytes with variable GRC numbers [2,10].

In bullfinch GRC bivalents, MLH1 signals occur at the H3K9me3-negative part, which is consistent with the known effect of heterochromatin in suppressing recombination [33,34]. The presence of a large H3K9me3-negative region in bullfinch GRCs enables pairing along their entire length, whereas in mosaic males of other species, their completely H3K9me3-positive GRCs form only end-to-end associations. It is plausible to suggest that pre-recombination events occurring in the H3K9me3-negative region of the bullfinch GRC facilitate the establishment and extension of the central element of the synaptonemal complex along the entire chromosome.

Unfortunately, the behavior of the GRC in bullfinch female meiosis has not yet been studied, so we cannot assess the degree of sexual dimorphism in this trait. Further comparative studies of the genetic composition of the GRC, its epigenetic modifications, variations in copy number, and its behavior in male and female meiosis are necessary to understand the structure, function, and evolution of this remarkable chromosome.

## 5. Conclusions

In this study, we demonstrated normal meiotic behavior of the GRC and the basic chromosome set within the rare tetraploid and octoploid spermatocytes of the bullfinch. Unlike in other species, where two supernumerary GRCs fail to pair completely in male meiosis, the bullfinch GRCs achieve full synapsis and spatially restricted recombination in euchromatic, H3K9me3-negative part of the GRC bivalent. The surprising normality of meiosis in these polyploid cells, with regular synapsis and the same recombination rates per bivalent comparable to diploids, suggests that they are not automatically arrested and could potentially progress to produce diploid sperm. This finding provides a plausible cytological mechanism for the generation of unreduced gametes, which are known to give rise to the rare but documented cases of viable, albeit sterile, triploid birds in various species.

## Figures and Tables

**Table 1:** The antibodies used in this study.

Antibody	Host	Supplier (Catalog no.)	Dilution	Reaction Type
Anti-SYCP3	Rabbit	Abcam (ab15093)	1:300	Unconjugated
Anti-centromere antibodies	Human	Antibodies Inc. (15-234)	1:70	Unconjugated
Anti-H3K9me3	Rabbit	Abcam (ab8898)	1:100	Unconjugated
Anti-MLH1	Mouse	Abcam (ab14206)	1:30	Unconjugated
Anti-mouse	Goat	Jackson ImmunoResearch (115-095-003)	1:30	FITC
Anti-rabbit	Goat	Jackson ImmunoResearch (111-165-144)	1:250	Cy3
Anti-human	Donkey	Jackson ImmunoResearch (709-155-149)	1:65	AMCA
Anti-rabbit	Donkey	Jackson ImmunoResearch (711-095-152)	1:100	FITC
Anti-rabbit	Goat	Jackson ImmunoResearch (111-175-144)	1:100	Cy5

## Data Availability

The data presented in this study are available in the article and Supplementary Material.

## Supplementary Materials

The following supporting information can be downloaded at: https://www.mdpi.com/article/10.3390/ani15233394/s1, Figure S1: Microphotographs of tetraploid bullfinch spermatocytes at zygotene, early pachytene, and mid-late pachytene stages after immunostaining with antibodies to SYCP3, centromere proteins, MLH1 and FISH with the GRC-specific probe; Figure S2: Microphotographs of tetraploid bullfinch spermatocytes after immunostaining with antibodies to SYCP3, centromere proteins, and MLH1; Table S1: Measurement data of GRC univalent in diploid cells, GRC bivalent in polyploid cells, tetraploid cells, and quadrivalents.
