# Supplementary material for: Germline-Restricted Chromosome (GRC) in Diploid and Polyploid Spermatocytes of the Eurasian Bullfinch, Pyrrhula pyrrhula (Fringillidae, Passeriformes, Aves)
Source: Animals (Basel). 2025 Nov 24;15(23):3394. doi: 10.3390/ani15233394 (PMC12691318; doi:10.3390/ani15233394)
Supplement: Supplementary file 1 [file animals-15-03394-s001.zip › Supplementary Figures.pdf]

Supplementary Material for:

Germline restricted chromosome (GRC) in diploid and polyploid spermatocytes of the Eurasian bullfinch, *Pyrrhula pyrrhula* (Fringillidae, Passeriformes, Aves)

Ekaterina Grishko [1,2], Lyubov Malinovskaya [1,2], Katerina Tishakova [1,3] and Pavel Borodin [2]*

[1] Laboratory of genome structure and function; Novosibirsk State University, 630090, Novosibirsk, Russia
[2] Laboratory of Recombination and Segregation Analysis, Institute of Cytology and Genetics, 630090 Novosibirsk, Russia
[3] Laboratory of Diversity and Evolution of Genomes, Institute of Molecular and Cellular Biology, 630090 Novosibirsk, Russia
* Correspondence: borodin@bionet.nsc.ru

This PDF file includes:

Figure S1 and S2

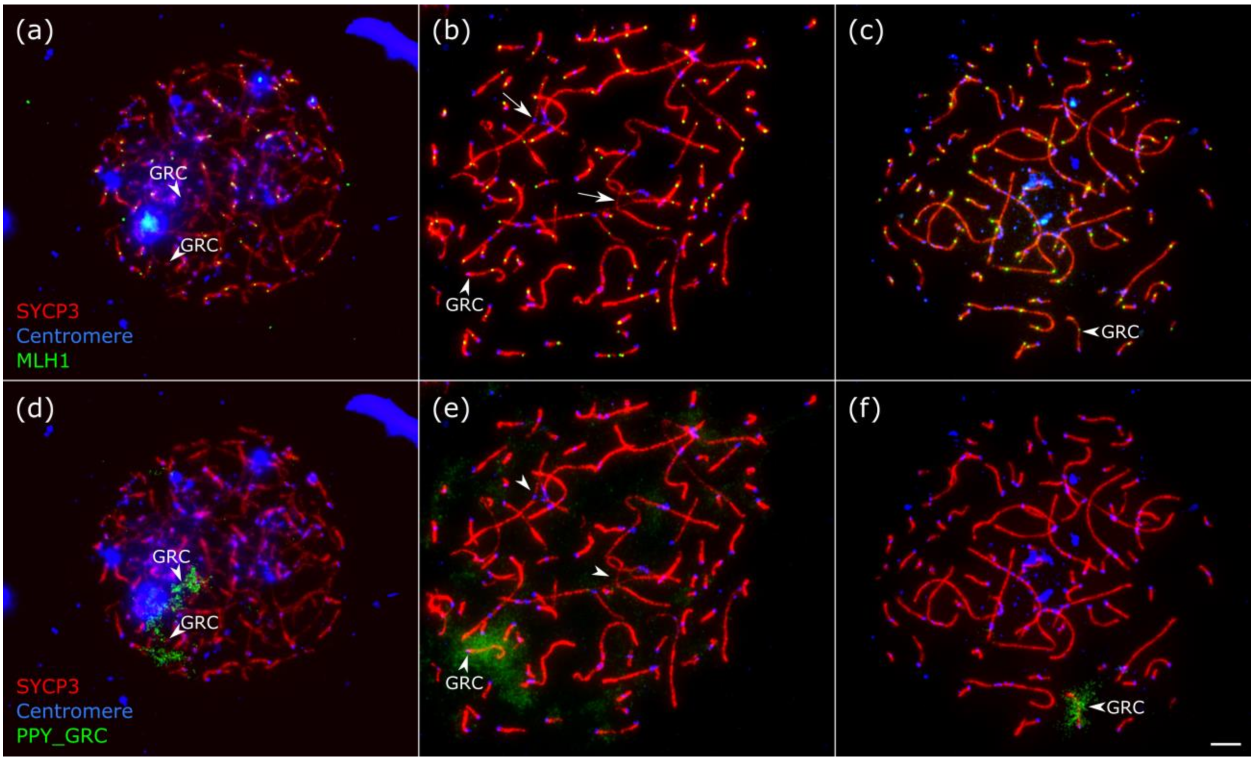

Figure S1. Microphotographs of tetraploid bullfinch spermatocytes at zygotene (**a**, **d**), early pachytene (**b**, **e**), and mid-late pachytene (**c**, **f**) stages after immunostaining with antibodies to SYCP3 (**a**-**f**), centromere proteins (**a**-**f**), MLH1 (**a**-**c**) and FISH with the GRC-specific probe (PPY_GRC) (**d**-**f**). Arrowheads indicate the GRC univalents (**a**, **d**) and bivalents (**b**, **c**, **e**, **f**). Arrows indicate quadrivalents (**b**, **e**). Scale bar - 5 μm.

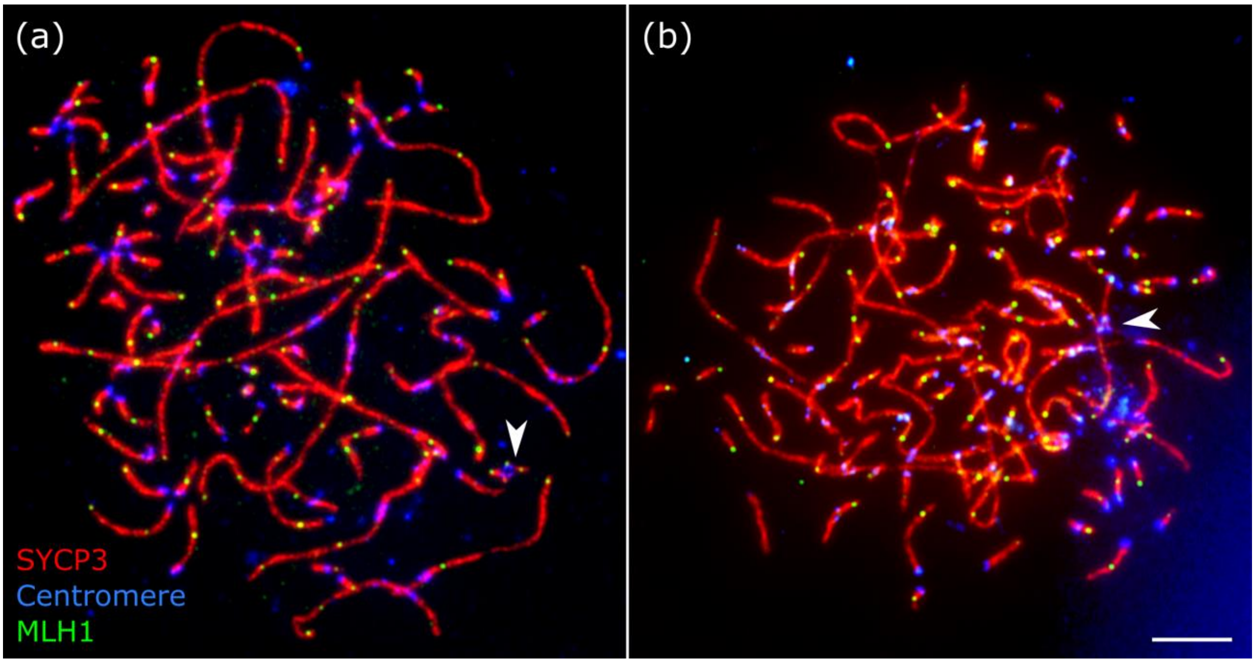

Figure S2. Microphotographs of tetraploid bullfinch spermatocytes after immunostaining with antibodies to SYCP3, centromere proteins, and MLH1. Arrowheads indicate quadrivalents. Scale bar - 5 μm.
